# Is Metformin a Possible Beneficial Treatment for Psoriasis? A Scoping Review

**DOI:** 10.3390/jpm11040251

**Published:** 2021-03-30

**Authors:** Ana Maria Alexandra Stanescu, Anca Angela Simionescu, Mira Florea, Camelia Cristina Diaconu

**Affiliations:** 1 Department of Family Medicine, “Carol Davila” University of Medicine and Pharmacy, 050474 Bucharest, Romania; alexandrazotta@yahoo.com; 2 Department of Obstetrics and Gynecology, Filantropia Clinical Hospital, “Carol Davila” University of Medicine and Pharmacy, 050474 Bucharest, Romania; 3 Community Medicine Department, Iuliu Hatieganu University of Medicine and Pharmacy, 400000 Cluj-Napoca, Romania; 4 Department of Internal Medicine, Clinical Emergency Hospital of Bucharest, “Carol Davila” University of Medicine and Pharmacy, 050474 Bucharest, Romania; drcameliadiaconu@gmail.com

**Keywords:** psoriasis, metformin, metabolic syndrome

## Abstract

Psoriasis is a chronic inflammatory condition with genetic, immunological, and metabolic etiology. The link between psoriasis and diabetes mellitus has been shown in genetic predisposition, environmental influences, inflammatory pathways, and insulin resistance, resulting in end-organ damage in both conditions. Because comorbidities often accompany psoriasis, the therapeutic management of the disease must also take into consideration the comorbidities. Given that metformin’s therapeutic role in psoriasis is not yet fully elucidated, we raised the question of whether metformin is a viable alternative for the treatment of psoriasis. We conducted this scoping review by searching for evidence in PubMed, Cochrane, and Scopus databases, and we used an extension for scoping reviews (PRISMA-ScR). Current evidence suggests that metformin is safe to use in psoriasis. Studies have shown an excellent therapeutic response to metformin in patients with psoriasis and comorbidities such as diabetes, metabolic syndrome, and obesity. There is no clear evidence supporting metformin monotherapy in patients with psoriasis without comorbidities. There is a need to further evaluate metformin in larger clinical trials, as a therapy in psoriasis.

## 1. Introduction

Diabetes mellitus is a chronic disease affecting over 22 million people worldwide and has metabolic, inflammatory, and pathological genetic mechanisms [1,2]. A first-line treatment in type 2 diabetes, 1,1-dimethyl biguanide hydrochloride (metformin), is a biguanide that reduces hyperglycemia, prevents inflammation, normalizes lipid and carbohydrate metabolism, and reduces adipose tissue [3,4].

The good results obtained with metformin as the first-line treatment of type 2 diabetes have led to its successful use in many other conditions, such as cancer (breast, endometrial, colorectal, prostate, various other tumors), nonalcoholic fatty liver disease, chronic kidney disease, metabolic syndrome, obesity, coronary artery disease, polycystic ovary syndrome, and acne. It also has anti-aging effects and improves the efficiency of in vitro fertilization; some studies have demonstrated the benefits of metformin in patients with psoriasis [5,6,7,8,9,10,11,12,13,14,15]. Psoriasis is a chronic inflammatory condition with genetic, immunological, and metabolic etiology that affects over 8 million people in the US [16,17]. It is considered a severe, non-communicable disease [18]. Psoriasis is a systemic disease with numerous multiorgan complications because of chronic inflammation; due to the dominant TNF-α-IL-23-Th17 axis, chronic inflammation leads to dysfunctional differentiation, uncontrolled keratinocyte proliferation, and neovascularization [19]. Psoriasis and diabetes mellitus are linked by genetic predisposition, environmental influences, inflammatory pathways, and insulin resistance, which result in end-organ damage in both conditions [20]. By reducing hyperglycemia and metabolic syndrome, suppressing endogenous glucose production, metformin may play an important role in psoriasis [21]. There are currently several therapeutic options for psoriasis. These may vary by country, by associated comorbidities, and by severity of disease.

Topical treatment is used as first-line therapy or as a combination therapy depending on the severity of psoriasis. Among topical therapies used are corticosteroids, tar derivatives, calcineurin inhibitors, and vitamin D analogues [22,23]. Another therapeutic option is phototherapy with PUVA (psoralen and ultraviolet A) or UVB on its own or in combination with other therapies. Classical systemic therapy includes methotrexate, acitretin, and cyclosporine, but also other systemic therapies represented by biological agents. Even with these wide therapeutic options, psoriasis cannot always be controlled [22,24,25].

Because psoriasis treatment can be a challenge in the medical practice, there is an openness to new therapeutic approaches that have not been considered so far, such as oral administration of vitamin D or metformin [26]. Given that metformin’s therapeutic role in psoriasis is not yet fully elucidated, we raised the question of whether metformin could be a viable alternative for the treatment of psoriasis. We conducted a scoping review about the utility of metformin in patients with psoriasis. We searched to identify the current evidence and determine the gaps that will lead to new research.

## 2. Materials and Methods

### 2.1. Search Strategy

For this scoping review, we used the adapted Preferred Reporting Items for Systematic reviews and Meta-Analyses (PRISMA) checklist and the extension for scoping reviews (PRISMA-ScR) [27,28]. For the literature search, we used the PubMed, Cochrane, and Scopus databases, for articles published between November 2020 and January 2021. We searched these databases using the following search terms: “psoriasis” AND “metformin”.

### 2.2. Inclusion and Exclusion Criteria

We scanned all the articles included in the research area. We included articles in English that refer to metformin treatment in patients with psoriasis (regardless of the form or severity of psoriasis). Because the number of search results was small, we did not apply filters regarding year of publication, gender, age, or type of study. Articles that analyzed the mechanism and effectiveness of metformin on human and animal skin cell cultures and those with case-control studies and randomized studies of administration of metformin were included. We did not exclude combination therapies or patients with comorbidities. We excluded articles in which patients were not diagnosed with psoriasis or in which there was no reference to metformin in the context of psoriasis.

## 3. Results

**Figure 1:**
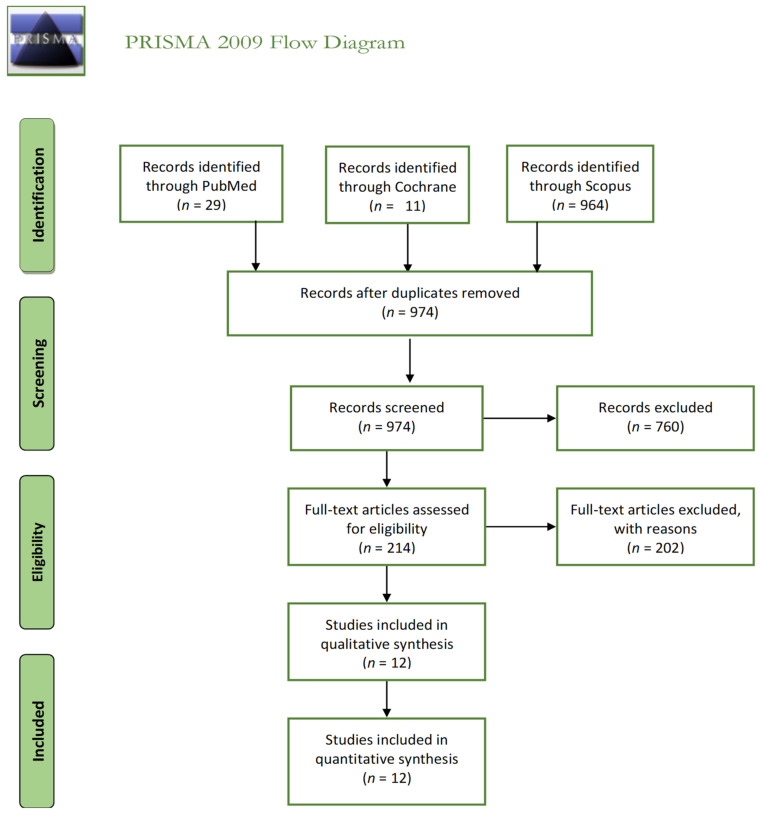
The Preferred Reporting Items for Systematic reviews and Meta-Analyses (PRISMA) flow diagram.

We used the PRISMA principles to develop this review. The PRISMA flow diagram is shown in Figure 1. We identified a total of 1004 records; after removing the duplicates, 974 articles remained. After screening and full-text assessment, we included 12 articles for further analysis in this scoping review.

We found six ongoing studies for which we did not have access to preliminary data. A minimal number of studies have addressed the mechanism of action, administration, and safety of metformin in psoriasis. Next, we present an overview of the evidence regarding the benefits and risks of this therapeutic option that has been scarcely studied so far.

### 3.1. Evidence from Experimental and Skin Cell Culture Studies Supporting the Use of Metformin for Psoriasis

Metformin may relieve the symptoms of hyperplastic epidermal diseases. As reported, metformin inhibited human immortalized keratinocyte (HaCaT) cell proliferation by downregulating the AMP-activated protein kinase (AMPK) and Erk signaling pathways. It appears that metformin can inhibit proinflammatory cytokines and induce HaCaT cell apoptosis, possibly by inhibiting mTOR signaling [29]. More recently, it has been shown that metformin both decreases HaCaT cell proliferation and induces HaCaT cell apoptosis in a dose-dependent manner, consistent with the effects of metformin in psoriasis; further, reactive oxygen species play a central role in the MET-triggered apoptosis of HaCaT cells [30,31]. All these effects pave the way for metformin as a possible treatment for psoriasis.

Metformin activates the enzyme AMPK, which suppresses inflammatory responses by downregulating the activity of dendritic cells, T-lymphocytes, macrophages, endothelial cells, and monocytes. By inhibiting complex I (NADH-ubiquinone-reductase), located in the inner mitochondrial membrane, metformin reduces reactive oxygen species formation and can profoundly alter T-cell responses [21,32,33,34]. Table 1 presents a summary of studies of metformin’s effects on skin cultures.

All these effects have been reconfirmed and emphasized by recent studies claiming that metformin significantly decreases the mRNA and protein levels of tumor necrosis factor alpha (TNFα), interleukin (IL)-6, IL-8, and IL-1β induced by TNFα and inhibits the nuclear localization of p65, a subunit of nuclear factor kappa B (NF-κB) [37].

### 3.2. Evidence from Clinical Studies Supporting Add-On Metformin for Psoriasis

From another perspective, namely methotrexate treatment in patients with psoriasis and psoriatic arthritis, for which it is commonly prescribed, metformin has been shown to improve drug-related liver toxicity [38]. From the perspective of quality of life, combined therapy with metformin and methotrexate significantly improves the quality of life of patients with psoriasis compared with therapy with methotrexate alone [39]. Combination therapy with metformin and methotrexate has also been shown to be more effective in treating psoriatic arthritis than methotrexate alone, with a more potent anti-inflammatory effect [40]. There are many similarities between metformin and methotrexate regarding biokinetics and dose dependence [21,41,42].

Psoriasis is an inflammatory disease. A study by Kim et al. evaluated the incidence of autoimmune diseases in diabetic patients by administering the combined therapy of a dipeptidyl peptidase-4 inhibitor (DPP4i) (second or third intention therapy in type 2 diabetes) and metformin (first-line therapy in type 2 diabetes) versus non-DPP4i and metformin combination therapy. The results suggested that the use of DPP4i combination therapy with metformin decreased the risk of autoimmune diseases in these patients, opening new pharmacological pathways for targeting these diseases [43]. Table 2 includes studies claiming that metformin is beneficial in the prevention of psoriasis.

Patients with diabetes are at risk of developing psoriasis. Diabetic patients have been shown to have a reduced risk of psoriasis if they use metformin frequently compared with those who use it occasionally. On the other hand, patients who have a history of insulin treatment, although frequent users of metformin, do not have a lower risk of psoriasis [44].

One randomized placebo-controlled study published in 2016 was the first to measure psoriasis progression in patients with metabolic syndrome; this study showed that metformin causes a significant improvement in Psoriasis Area Severity Index (PASI) score compared to placebo, and also a complete improvement in metabolic syndrome. The same study found a decrease in cardiovascular risk factors (lipid profile and blood pressure) that may contribute to a decrease in diabetes and cardiovascular mortality in psoriasis patients with metabolic syndrome [45].

The same authors published a similar study in 2017; they followed patients with psoriasis and metabolic syndrome after metformin administration in a dose of 1000 mg/day. After treatment with metformin, there was a reduction of the mean PASI, Erythema, Scaling, and Induration (ESI), and Physician Global Assessment (PGA) scores, and 45% of patients had complete improvement. Patients taking metformin had a significant decrease in body weight, body mass index (BMI), waist circumference, serum triglycerides, total cholesterol, and low-density lipoprotein cholesterol (LDL-C) [46].

A case-control study supports the hypothesis of a possible beneficial effect of thiazolidinediones in psoriasis and a decreased risk of a first-time psoriasis diagnosis in current users of ≥5 thiazolidinedione prescriptions and also found a possible reduced risk for current metformin use of ≥15 prescriptions [42].

In a recent report, 10 injections of itolizumab 1.6 mg·kg^−1^ administered to a patient with psoriasis and metabolic syndrome resulted in a decreased PASI score. Subsequent treatment included weight loss and metformin 500 mg twice daily, decreasing the PASI score from 30.7 in the 18th to 22.2 in the 21st month [47].

A rare case of type B insulin resistance in association with mixed connective-tissue disease and psoriasis has been described by Łebkowska et al. Targeted, individualized therapy with a combination of metformin, hydroxychloroquine, and methotrexate proved effective [48]. Moore et al. presented the case of a patient with psoriasis who worsened with the introduction of insulin therapy. Stopping insulin and reintroducing metformin led to the remission of psoriasis [49].

El-Gharabawy et al. followed psoriasis patients under topical treatment (coal tar, vitamin D3 analogues, and corticosteroids) and oral metformin 850 mg twice daily. Treatment with the anti-psoriatic drug combined with metformin and pioglitazone resulted in a significant decrease in CD4+ T cells, but no significant change in CD8+ T cells was detected. The same study showed that treatment of patients with anti-psoriatic drug alone or in combination with metformin caused a significant decrease in serum IL-2 compared with patients with psoriasis who did not receive treatment, with serum C-reactive protein also declining in patients treated in combination with metformin [50]. The current evidence for metformin benefits in psoriasis patients is shown in Table 3.

### 3.3. Safety of Metformin Treatment in Psoriasis

No increased mortality, severe psoriasis, psoriasis-related admission, or any other cause of admission was found in an observational study of 2277 patients with psoriasis and diabetes who received metformin when compared with a group of the same size without metformin. The safety of metformin is generally accepted for patients with psoriasis, even those with comorbidities such as coronary artery disease, hypertension, and dyslipidemia [51]. Metformin use may be a concern in patients with renal failure, as it is contraindicated in chronic kidney disease [52,53].

In addition to its role in psoriasis, metformin may play an essential role in several dermatological conditions such as acanthosis nigricans, acne, disorders of increased pigmentation, allergic contact dermatitis, eruptive xanthomas, suppurative hidradenitis, hirsutism, squamous cell carcinoma, and melanoma [54,55,56]. Several antidiabetic drugs have been studied for their effect on psoriasis (Table 4).

### 3.4. Evidence against Metformin Treatment in Patients with Psoriasis

Koca et al. presented a case with a psoriasiform eruption that appeared one week after the initiation of treatment with metformin, in a patient under no other treatment. The lesions disappeared 5 weeks after stopping treatment and reappeared after 4 months, when metformin was reintroduced, suggesting that metformin is likely to induce psoriasiform eruptions [65].

**Figure 2:**
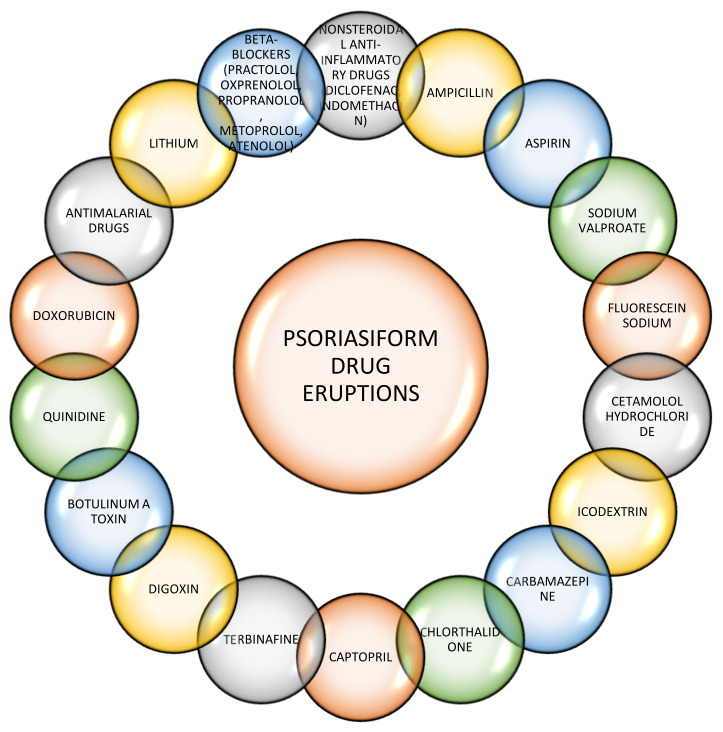
Several drugs have been implicated in psoriasiform drug eruptions, trigger new-onset psoriasis, or exacerbate existing psoriasis [67,68,69,70,71,72,73,74].

Another case, reported by Voore et al., was of a patient with known psoriasis who was later diagnosed with diabetes, started treatment with metformin and, at 2 weeks, presented with drug reaction with eosinophilia and systemic symptoms (DRESS) syndrome (diagnosed based on the presence of three of the five symptoms described in the RegiS-CAR criteria for the diagnosis of the DRESS syndrome). DRESS syndrome resolved upon discontinuation of metformin [66]. Other drugs are known to trigger psoriasiform drug eruptions (Figure 2).

## 4. Discussion

The biguanide metformin is more than an antidiabetic drug; it has a direct anti-inflammatory effect. The anti-inflammatory mechanism of action of metformin is based on AMPK activation and inhibition of mTOR pathways. Metformin acts on mitochondrial function and cellular homeostasis processes; dysregulation of autophagy or mitochondrial function in immune cells raises the susceptibility to develop autoimmune and inflammatory diseases [75].

Chronic inflammation is essential in the pathophysiology of psoriasis, being a predisposing factor for the development of other diseases, such as diabetes. Patients with psoriasis have a higher risk of developing abdominal obesity, insulin resistance, and dyslipidemia, leading to the development of metabolic syndrome [76]. Due to predisposition to obesity of patients with psoriasis and systemic inflammation, both may contribute to the development of insulin resistance and type 2 diabetes in these patients [77].

Osteopontin participates in inflammation, being secreted by T-lymphocytes and activated by macrophages; low levels of osteopontin or lack of expression reduce inflammation [78]. In psoriasis, osteopontin acts by promoting vessel formation, subsequently supporting the influx of inflammatory cells through a mechanism mediated by IL-1 and matrix metalloproteinase-9 [79].

It is considered that osteopontin is involved in the development of insulin resistance, obesity, and type 2 diabetes [80]. Metformin attenuates the upregulation of osteopontin and monocyte chemoattractant protein 1 induced by oxalate in vitro. Metformin may simultaneously regulate these two molecular targets during the formation of stones [81].

Other mechanisms may influence the positive response to metformin therapy in patients with psoriasis associated with diabetes or metabolic syndrome, especially given the common role of inflammation.

Summarizing the evidence on metformin treatment in psoriasis, we can conclude that:

1. Metformin plays an important role in the treatment of autoimmune diseases.
2. Better results are achieved with metformin and methotrexate combined, compared with methotrexate therapy alone.
3. Good results have also resulted from topical therapy combined with metformin.
4. Metformin decreases the risk of developing psoriasis in diabetic patients.
5. Good results have been achieved with metformin treatment in patients with psoriasis and metabolic syndrome, for both psoriasis and metabolic syndrome.
6. Metformin is generally safe for administration in patients with psoriasis.

## 5. Conclusions

In this review, we demonstrated that metformin is safe to use in patients with psoriasis associated with diabetes, metabolic syndrome, and obesity. Antidiabetic agents may be useful for the treatment of psoriasis, especially with co-existing diabetes or when immunosuppression is contraindicated. Moreover, in psoriasis accompanied by metabolic syndrome with an inadequate response to biological therapies, metformin could be an alternative treatment and an important add-on in the management of this chronic autoimmune disease. Because comorbidities often accompany psoriasis, the therapeutic management of the disease must also take into consideration the comorbidities. There is a need to further evaluate metformin in larger clinical trials, as a therapy in psoriasis.

## Figures and Tables

**Table 1:** Skin culture studies included in the analysis.

Author	Year of Publication	Country	Type of Study	Mechanism and Effectiveness of Metformin Therapy in Psoriasis
Liu et al. [29]	2015	China	human keratinocytes HaCaT	- Metformin treatment significantly inhibited proliferation and proinflammatory responses (dose-dependently) in HaCaT cells, by a mechanism associated with inhibition of the mTOR signaling pathway. - Metformin inhibited the expression of IL-6, TNF-a, and VEGF proteins in HaCaT cells. - Metformin induced HaCaT cell apoptosis.
Wang et al. [30]	2018	China	human keratinocytes HaCaT	- Metformin could attenuate Raf-1-ERK1/2 signaling in HaCaT cells. - Metformin suppressed the expression and phosphorylation levels of Nrf2, which contributed to intracellular ROS generation and pro-apoptotic effects.
Wu et al. [31]	2017	China	Human HaCaT	- Metformin has antiproliferative and proapoptotic effects through the upregulation of ACAD10 expression, which is mediated by the negative regulation of mitochondria-mTORC1 signaling via the induction of cell-cycle arrest and apoptosis in human keratinocytes.
Ba et al. [35]	2018	China	Human cell culture	- Metformin significantly decreased the production of inflammatory cytokines and inhibited the nuclear localization of p65. - Metformin inhibits TNFα-induced inflammatory responses in HaCaT cells via nuclear factor kappa B (NF-κB) signaling.
				- Metformin suppresses the transcriptional activity of NF-κB by suppressing the degradation of IκBα. Furthermore, metformin’s inhibitory effect on NF-κB is comparable to that of the specific IKKβ inhibitor, BI605906.
Tsuji et al. [36]	2020	Japan	Animal tissue culture	- Metformin has immunomodulatory effects in an induced-psoriasis mouse model associated with type 2 diabetes mellitus. - TNF-α and IL-17A induce inflammatory responses by keratinocytes by blocking NLRP3 inflammasome activation in vitro. - Oral metformin treatment significantly attenuates IMQ-induced psoriasis-like inflammation in vivo. The therapeutic benefits can be partially attributed to its interference with mature IL-1β secretion by keratinocytes.

**Table 2:** Studies about the occurrence of psoriasis in other diseases.

Author	Year of Publication	Country	Type of Study	Diseases
Kim et al. [43]	2014	US	Cohort	Autoimmune diseases (rheumatoid arthritis, systemic lupus erythematosus, psoriasis, multiple sclerosis, and inflammatory bowel disease).
Wu et al. [44]	2015	Taiwan	Case-control	Diabetic patients, especially among those who have never used insulin.

**Table 3:** Studies on metformin administration in psoriasis patients.

Author	Year of Publication	Country	Type of Study	Metformin Therapy in Psoriasis	Dose
Brauchli et al. [42]	2008	Switzerland	Case-control	Effect of long-term use of metformin in obese patients associated with thiazolidinediones	
Singh and Bhansali [45]	2016	India	Randomized	Metformin improved features of metabolic syndrome (MS). Metformin and pioglitazone improving MS parameters might account for the improved efficacy in psoriasis itself. The anti-proliferative and anti-inflammatory action of metformin might have resulted in improvement of the psoriasis. There is significant reduction in weight with the use of metformin, and due to controversy about increased risk of bladder cancer associated with pioglitazone, metformin can be preferred over pioglitazone in psoriasis patients with MS.	Metformin 1000 mg once daily (O.D) or pioglitazone 30 mg
Singh et al. [46]	2017	India	Randomized	Metformin has shown improvement in psoriasis and parameters of MS; hence, it can be used for the benefit of psoriasis patients having MS. The metformin group had greater percentage reduction in mean PASI, ESI, and PGA scores as compared with placebo. In total, 45% of the patients had complete improvement in MS in the metformin group as compared with 33.3% of patients in the placebo group. Patients taking metformin had statistically significant decreased weight, BMI, waist circumference, fasting plasma glucose, serum triglycerides, total cholesterol, and LDL-C as compared with patients taking placebo.	
El-Gharabawy et al. [50]	2016	Saudi Arabia	Randomized Study	Metformin modulates the immune system (causing a significant decline in CD4+ T cells) in psoriasis and MS or impaired glucose tolerance and has a remarkable effect in the early stages of psoriasis. Therefore, either pioglitazone or metformin in combination with traditional anti-psoriatic drugs provides better results in the treatment of psoriasis than does either alone.	850 mg twice daily
Su et al. [51]	2019	Taiwan	Retrospective cohort study 1995–2014	Metformin can be prescribed for diabetic psoriasis patients without chronic kidney disease.	

**Table 4:** Antidiabetic drugs used in patients with psoriasis [45,46,57,58,59,60,61,62,63,64].

Antidiabetic Drug	Dose	Period	Country	Year	Authors
Metformin	1000 mg	12 weeks	India India	2016 2017	Singh and Bhansali [45] Singh and Bhansali [46]
Pioglitazone	15–30 mg	10–16 weeks	India India India Iran Egypt Iran	2016 2009 2005 2019 2015 2015	Singh and Bhansali [45] Mittal at al. [58] Shafiq et al. [59] Ghiasi et al. [60] Hafez et al. [61] Lajevardi et al. [62]
Rosiglitazone	2–8 mg	26 weeks	SUA	2007	Ellis et al. [63]
Liraglutide subcutaneous injection	Initial dose: 0.6 mg—1 week 1.2 mg—1 week 1.8 mg—6 weeks	8 weeks	Denmark	2015	Faurschou et al. [64]
